# The Pleiotropic Effects of GATA1 and KLF1 in Physiological Erythropoiesis and in Dyserythropoietic Disorders

**DOI:** 10.3389/fphys.2019.00091

**Published:** 2019-02-12

**Authors:** Gloria Barbarani, Cristina Fugazza, John Strouboulis, Antonella E. Ronchi

**Affiliations:** ^1^Dipartimento di Biotecnologie e Bioscienze, Università degli Studi Milano-Bicocca, Milan, Italy; ^2^School of Cancer & Pharmaceutical Sciences, Faculty of Life Sciences & Medicine, King’s College London, London, United Kingdom

**Keywords:** erythropoiesis, dyserythropoiesis, transcription factors, GATA1, KLF1

## Abstract

In the last few years, the advent of new technological approaches has led to a better knowledge of the ontogeny of erythropoiesis during development and of the journey leading from hematopoietic stem cells (HSCs) to mature red blood cells (RBCs). Our view of a well-defined hierarchical model of hematopoiesis with a near-homogeneous HSC population residing at the apex has been progressively challenged in favor of a landscape where HSCs themselves are highly heterogeneous and lineages separate earlier than previously thought. The coordination of these events is orchestrated by transcription factors (TFs) that work in a combinatorial manner to activate and/or repress their target genes. The development of next generation sequencing (NGS) has facilitated the identification of pathological mutations involving TFs underlying hematological defects. The examples of GATA1 and KLF1 presented in this review suggest that in the next few years the number of TF mutations associated with dyserythropoietic disorders will further increase.

## Introduction

Erythropoiesis leads to the production of the proper number of RBCs required by the body under homeostatic and stress conditions. In healthy adults, erythropoiesis ensures the release in the blood stream of 2 × 10^6^ RBCs/second, but this number dramatically increases to respond to inadequate tissue oxygenation (Tsiftsoglou et al., 2009; Dzierzak and Philipsen, 2013; Nandakumar et al., 2016).

Insufficient quantitative or qualitative production of fully functional RBCs, whether acquired or inherited, results in a wide spectrum of diseases generally defined as anemias.

The causes of anemias are variable and reflect the complexity of the differentiation and maturation of erythrocytes. In some cases, the number of RBCs is extremely low because of the failure to produce erythroid progenitors, as in Diamond-Blackfan Anemia (DBA) (Da Costa et al., 2018). In other cases, impaired differentiation leads to the accumulation of erythroid precursors in the bone marrow [β-thalassemia (Rivella, 2015), congenital dyserythropoietic anemia, CDA (Iolascon et al., 2011)] or to the unbalanced production of different blood cell types [myelodysplastic syndromes, MDS (Levine et al., 2007; Lefevre et al., 2017)], resulting in insufficient RBC numbers in the bloodstream. In other forms of anemias, RBCs are produced but defects in some crucial gene products [typically specific enzymes (Koralkova et al., 2014; Grace et al., 2018), membrane proteins or cytoskeletal components (Mohandas and Gallagher, 2008; Perrotta et al., 2008), sickle globin chains (Rees et al., 2010), channel proteins (Glogowska and Gallagher, 2015), specific pathways (Bianchi et al., 2009; Schwarz et al., 2009)] result in RBCs with decreased oxygen delivery capacity and/or shortened lifespan. Very often, different diseases share common features: for example imbalanced globin chains in β-thalassemia is accompanied by the accumulation of defective precursors in the bone marrow and by ineffective erythropoiesis (IE), as is also observed in CDA (Libani et al., 2008; Iolascon et al., 2011; Ribeil et al., 2013; Rivella, 2015).

Recently, thanks to the advent of new technologies, including NGS using small pools of cells or single cells (Nestorowa et al., 2016; Paul et al., 2016; Ye et al., 2017; Giladi et al., 2018), the development of improved panels of surface markers (Guo et al., 2013; Notta et al., 2016) and the design of *in vivo* cell tracing systems (Dykstra and Bystrykh, 2014; Perie et al., 2014; Pei et al., 2017; Rodriguez-Fraticelli et al., 2018; Upadhaya et al., 2018), our understanding of hematopoiesis -and erythropoiesis- has greatly expanded. In parallel, genome wide association approaches (GWAS) (Menzel et al., 2007; Sankaran et al., 2008; Uda et al., 2008; Soranzo et al., 2009; van der Harst et al., 2012), massive genome and exome sequencing (Chami et al., 2016) led to the identification of new variant/modifier alleles influencing erythropoiesis associated with TFs.

In this scenario, TFs not only control lineage commitment transitions but are emerging as key-players underpinning, so far unexplained erythroid diseases. Here, we consider GATA1 and KLF1 as paradigmatic TFs. By focusing on these examples, we aim to provide evidence of their pleiotropic effects rather than to give a complete list of GATA1 or KLF1 mutations identified so far.

## Erythropoiesis

### Erythropoiesis During Development

The first wave of erythropoiesis originates in the yolk sac, where Primitive Erythroid Cells (EryPs) sustain the oxygenation demand of the growing embryo (Dzierzak and Philipsen, 2013). EryPs are large in size and still nucleated when released in the circulation, where they later enucleate (Isern et al., 2011; Dzierzak and Philipsen, 2013; Palis, 2014). In mouse, at E8.25 a second wave of erythro-myelo-precursors (EMPs) originates in the yolk sac and colonizes the fetal liver, generating the first definitive RBCs (Palis, 2016). Finally, around E10.5, hematopoietic stem cells (HSCs) from aorta-gonad-mesonephros (AGM), placenta and possibly other yet unknown sites, colonize the fetal liver. These cells will sustain definitive hematopoiesis for the remainder of gestation and, around birth, will migrate to the bone marrow, the site of adult hematopoiesis (Dzierzak and Philipsen, 2013).

### From HSC to RBC

Until recently, the “classical model” of hematopoiesis was considered a paradigm of a stepwise, hierarchical cellular specification system, whereby HSCs generated multipotent progenitors with progressively restricted lineage potential through a sequence of binary choices. The grand entrance of new single-cell separation technologies, *in vivo* lineage tracing systems and single-cell analysis, provided novel and surprising insights, prompting the idea that early transcriptional priming develops into the acquisition of specific lineage programs (Cabezas-Wallscheid et al., 2014; Haas et al., 2018). In this context, erythroid cells would originate early in the hematopoietic hierarchy, i.e., from stem/multipotential progenitor stages (Guo et al., 2013; Notta et al., 2016; Tusi et al., 2018), soon after the emergence of the megakaryocytic lineage (Upadhaya et al., 2018).

The first clearly recognizable unipotent erythroid progenitor, identified decades ago in *in vitro* clonogenic assays, is the BFU-E (burst-forming unit-erythroid), that differentiate into rapidly dividing colony-forming-unit erythroid (CFU-E) (Hattangadi et al., 2011; Koury, 2016; Dulmovits et al., 2017). The entry of CFU-Es into erythroid terminal differentiation marks the transition into final maturation (Hwang et al., 2017; Tusi et al., 2018).

## Extracellular and Intracellular Signals

**FIGURE 1:**
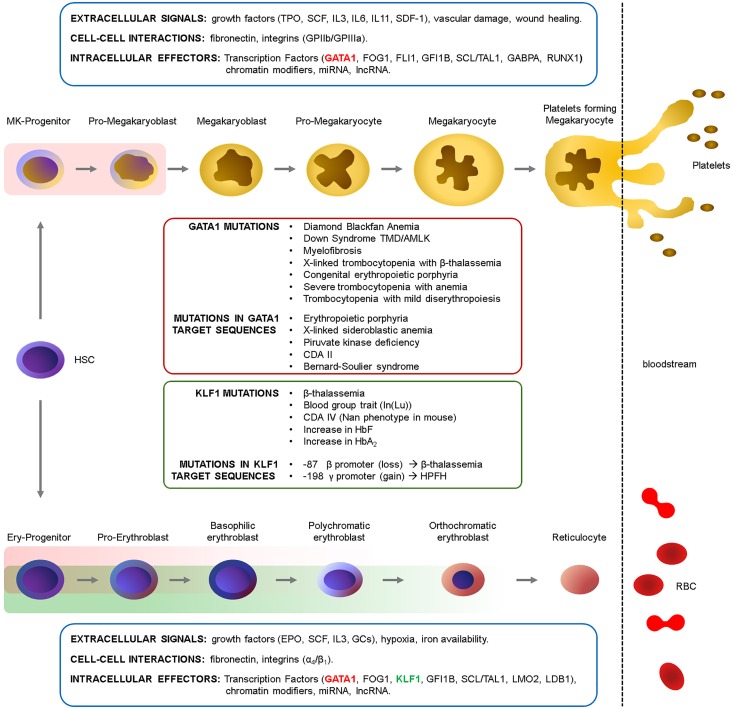
Erythropoiesis and megakaryopoiesis are regulated at multiple levels. A complex network of extracellular signals -activating intracellular signaling pathways-, cell–cell interactions within the niche and intracellular effectors regulate cell differentiation in homeostatic conditions and in response to stress stimuli (Ferreira et al., 2005; Hattangadi et al., 2011; Songdej and Rao, 2017). These signals converge on TFs and chromatin modifiers which ultimately define the transcriptome at each given stage. The main growth factors, integrins and transcription factors involved in these processes are indicated. The GATA1 (red rectangles) and KLF1 (green rectangles) windows of expression are indicated (see also Figure 2B). HSC, Hematopoietic Stem cell; TPO, thrombopoietin; SCF, Stem cell Factor; IL, interleukin; SDF-1, stromal-derived factor-1; GPIIb/IIIa, integrins α_IIb_/β_3_ (CD41/CD61); EPO, erythropoietin; GCs, glucocorticoids. α_4_/β_1_, integrins α_4_/β_1_ (CD49d/CD29).

Red blood cell differentiation, their production in homeostatic and stress condition, is governed by an integrated complex interplay of extracellular and cell-cell signals within the microenvironment that activate the appropriate downstream intracellular signals, ultimately converging on key TFs. Although these aspects are beyond the scope of this review, we give a glimpse of the major players in these regulatory networks in Figure 1.

## The Role of Transcription Factors

**FIGURE 2:**
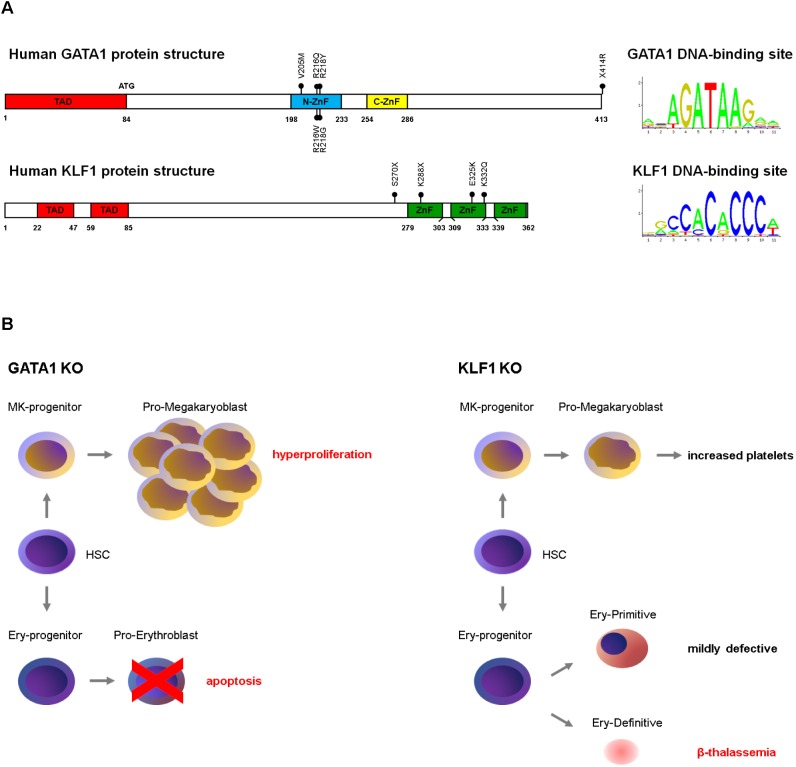
**(A)** Schematic structure of GATA1 and KLF1 proteins and of their DNA-binding motifs. The position of the mutations discussed in this review are indicated. ZnF, zinc fingers; TAD, transactivation domains. The DNA consensus are from the JASPAR database (http://jaspar2016.genereg.net/). **(B)** Phenotype of *GATA1* (Pevny et al., 1991, 1995; Fujiwara et al., 1996; Shivdasani et al., 1997; Gutierrez et al., 2008) and *KLF1* (Nuez et al., 1995; Perkins et al., 1995; Hodge et al., 2006; Nilson et al., 2006; Frontelo et al., 2007; Tallack and Perkins, 2010) gene knockouts in mouse.

Transcription factors, together with cofactors and chromatin modifiers, dictate the lineage-specific, stage-specific transcriptional programs by coordinately activating and/or repressing their targets through their binding to DNA (Portela and Esteller, 2010; Dore and Crispino, 2011; Love et al., 2014). The advent of NGS has rapidly expanded our understanding of TFs functions in physiological erythropoiesis, discovering TF mutations as cause of yet unexplained hematological -and dyserythropoietic- defects. Here, we focus on the key examples of GATA1 and KLF1 and their mutations to provide a glimpse of the complexity of their actions (Figure 2).

### The Example of the “Master Regulator” GATA1

The X-linked *GATA1* gene encodes a zinc finger TF expressed in the hematopoietic system in erythroid, megakaryocytic and, at lower levels, in eosinophilic, dendritic, and mast cells (Yu et al., 2002a; Ferreira et al., 2005; Gutierrez et al., 2007; Kozma et al., 2010).

GATA1 has three main functional domains: an N-terminal activation domain (N-TAD) and two homologous zinc (Zn) finger domains in the C-terminal half of the protein. The N-terminal Zn finger binds to the GATA1 main cofactor FOG1 (Friend-of-GATA) and modulates the affinity of GATA1 for binding to complex sites *in vitro* (Trainor et al., 1996; Newton et al., 2001; Yu et al., 2002b). The C-terminal Zn finger (C-ZnF) binds to DNA (WGATAR motif).

*GATA1* produces two isoforms: the full length protein (GATA1-FL, 47 kDa) and a shorter variant (GATA1s, 40 kDa), translated from codon 84 within the third exon. GATA1s lacks the N-TAD and results in a protein with a reduced transactivation activity (Calligaris et al., 1995). *Gata1* knockout in mice (Pevny et al., 1991) results in embryonic lethality around E10.5–E11.5 due to severe anemia, with GATA1-null cells undergoing massive apoptosis at the proerythroblastic stage (Pevny et al., 1995; Fujiwara et al., 1996). The conditional erythroid knockout in adult mice causes aplastic anemia, revealing its essential role in both steady-state and stress erythropoiesis (Gutierrez et al., 2008).

By contrast, megakaryoblasts lacking GATA1 proliferate abnormally but fail to undergo terminal differentiation (Shivdasani et al., 1997; Vyas et al., 1999). Since these first studies, many other reports revealed the many roles of GATA1 in the erythro/megakaryocytic differentiation (Ferreira et al., 2005). *GATA1* mutations identified in patients underscore this pleiotropy: mutations altering the quantity or quality of GATA1 can lead to a variety of phenotypes. Depending on the type of mutation and whether germline or somatic, the severity of the disease and the involvement of the erythroid and/or megakaryocytic compartments greatly varies.

## “Quantitative Mutations”: Gene Dosage and Background Effects at Work

### Mutations Causing GATA1-FL Loss: Inherited

Diamond-Blackfan anemia (DBA) is an inherited bone marrow failure syndrome characterized by severe anemia due to a great reduction in BFU-Es, without involvement of other hematopoietic lineages. Heterozygous mutations in ribosomal proteins account for about 65% of DBA cases. In 2012 an exome sequencing approach discovered the first *GATA1* mutation in a DBA patient (Sankaran et al., 2012). This mutation (c.220G > C transversion) causes the skipping of exon 2, determining GATA1-FL loss, while retaining GATA1s. Unrelated DBA patients were reported to carry the same mutation (Klar et al., 2014), or mutations in the ATG of GATA1-FL (Ludwig et al., 2014; Parrella et al., 2014). Of interest, in a family reported by Hollanda et al. (2006) the inherited loss of GATA1-FL results in macrocytic anemia of various severity in the different patients (with variable involvement of megakaryocytes and neutrophils).

### Mutations Causing GATA1-FL Loss: Acquired

Somatic mutations in *GATA1*, preventing the synthesis of GATA1-FL, predispose newborn Down Syndrome (DS) patients to develop (in 10–20% of cases) transient myeloproliferative disease (TMD) (Wechsler et al., 2002; Xu et al., 2003; Hitzler and Zipursky, 2005). This pre-leukemic condition often spontaneously resolves. However, in about 30% of TMD cases, it develops into acute pediatric megakaryoblastic leukemia (AMKL) (Wechsler et al., 2002; Magalhaes et al., 2006). All the DS-TMD *GATA1* mutations identified so far, map in exon 2 and either introduce a STOP codon or alter splicing such that only GATA1s is translated (Mundschau et al., 2003; Rainis et al., 2003). The loss of GATA1-FL in pre-malignant cells characterizes virtually all cases of DS-TMD. The detection of clone-specific *GATA1* mutations in DS-TMD and AMKL proves that AMKL derive from the TMD clone (Rainis et al., 2003; Ahmed et al., 2004; Hitzler and Zipursky, 2005). Moreover, *GATA1* mutations are extremely rare in AMKL blasts of non-DS patients, clearly indicating a specific cooperation of *GATA1* mutations with trisomy 21 (Gruber and Downing, 2015). The restoration of GATA1-FL expression in DS-AMKL-derived cells partially restores erythroid differentiation, further supporting the notion that the loss of GATA1-FL is essential for leukemogenesis (Xu et al., 2003). Importantly, DS-AMKL *GATA1* mutations have very little effect on erythropoiesis, suggesting that the co-occurrence trisomy 21 confers the property of specific targeting megakaryoblasts in DS patients.

Various evidences suggest that TMD likely emerges in a yolk sac/fetal liver progenitor *in* *utero* (Shimada et al., 2004). In agreement with this hypothesis, in mouse, a knockin allele abolishing GATA1-FL (and leaving GATA1s intact) results in a transient reduction of erythroid cells accompanied by increased megakaryopoiesis that resolves around E14.5 (Li et al., 2005). Despite these observations, the fetal cell type originating TMD and molecular mechanisms by which *GATA1* mutations specifically synergizes with trisomy 21 are still unclear (Crispino, 2005).

### GATA1 Low Levels and Disease

The notion that low levels of GATA1 lead to the development of myelofibrosis comes from studies in the GATA1-low mouse model, that also develops anemia with age (Vannucchi et al., 2002). In line with this first observation, the majority of patients with primary myelofibrosis (PMF) have GATA1-deficient megakaryocytes (Migliaccio et al., 2005). Of interest, in PMF patients, the reduced level of GATA1 is due to its impaired translation secondary to RPS14 deficiency (Gilles et al., 2017). The connection between GATA1 levels and RP proteins hinges on additional observations: indeed, in cells from DBA patients who are haploinsufficient for RPS19, GATA1 translation is greatly reduced (Ludwig et al., 2014; O’Brien et al., 2017; Khajuria et al., 2018).

Together, these examples again point toward the importance of the correct GATA1 protein dosage and indicates *GATA1* post-transcriptional regulation as an important determinant of GATA1 protein level.

## “Qualitative Mutations”: the Importance of Protein-Protein Interactions and More

### Mutations Abolishing the Interaction With FOG1

In Tsang et al. (1997) identified by yeast two-hybrid a novel zinc finger protein, named FOG1, binding to the N-ZnF of GATA1. GATA1 mutants unable to bind FOG1 (but still retaining DNA binding) do not rescue the severe block in terminal erythroid maturation of GATA1-deficient cells (Tsang et al., 1997). Instead, a compensatory FOG1 mutation restoring the interaction, rescues the GATA1^-^ phenotype, demonstrating that the interaction between the two proteins is essential for erythroid and megakaryocytic differentiation (Crispino et al., 1999; Chang et al., 2002). In Nichols et al. (2000) described a family with dyserythropoietic anemia and thrombocytopenia caused by a GATA1 (V205M) mutation abolishing the GATA1:FOG1 interaction.

### Other Allelic Variants, Other Interactions, Other Phenotypes

Remarkably, distinct substitutions at a single residue lead to very different outcomes, underlying the complexity of the GATA1 networks. The R216Q substitution causes X-linked thrombocytopenia with β-Thalassemia (Yu et al., 2002b; Balduini et al., 2004), whereas R216W patients also show features of congenital erythropoietic porphyria (CEP) (Phillips et al., 2007; Di Pierro et al., 2015). The D218Y mutation causes severe thrombocytopenia with anemia (Freson et al., 2002), whereas the D218G substitution causes macrothrombocytopenia with mild dyserythropoiesis and no anemia (Freson et al., 2001; Mehaffey et al., 2001).

Notably, whereas the D218Y diminishes the FOG1:GATA1 interaction, the D218G and R216Q do not, but they rather impair GATA1 ability to recruit the TAL1 cofactor complex (Campbell et al., 2013).

## Mutations in the GATA1 DNA Target Sequences as a Cause of Human Erythroid Disorders

Ultimately, TFs elicit their function by binding to DNA motifs on their target genes. Thus, it is expected that mutations creating new -or disrupting- specific binding sites could have phenotypic consequences. Although these mutations remain very elusive, over the years an increasing number of cases has accumulated, implicating these polymorphisms as a source of disease. Such mutations have been associated with congenital erythropoietic porphyria (Solis et al., 2001), X-linked sideroblastic anemia (Campagna et al., 2014; Kaneko et al., 2014), pyruvate kinase deficiency (Manco et al., 2000), CDAII (Russo et al., 2017), Bernard–Soulier syndrome (Ludlow et al., 1996) or linked to erythroid trait variants such as δ-thalassemia (Matsuda et al., 1992) and blood groups (Tournamille et al., 1995; Nakajima et al., 2013; Oda et al., 2015; Moller et al., 2018). Interestingly, a mutation abolishing a GATA1 consensus in the *KLF1* promoter (see below), causes a reduction of KLF1, which in turn results in reduced transcription of the KLF1 target genes more sensitive to KLF1 levels, such as *BCAM*, encoding for the Lutheran (Lu) antigen (Singleton et al., 2008).

### E/KLF1: An Unsuspected Key-Player in Various Types of Dyserythropoiesis

*KLF1* gene, located on chromosome 19, encodes for a proline-rich protein containing three zinc fingers (Bieker, 1996; Mas et al., 2011; Figure 1B), expressed in the bone marrow and in the erythroid lineage. KLF1 mainly acts by recruiting coactivators and chromatin remodelers, thus contributing to the large epigenetics changes which shape erythroid maturation (Shyu et al., 2014).

As for GATA1, the first evidence for an essential role in erythropoiesis came from the observation that *KLF1* knockout mice die *in utero* around E15 due to fatal anemia (Nuez et al., 1995; Perkins et al., 1995). Given that KLF1 is an important activator of β-globin, lethality was first attributed to β-thalassemia. However, this is not the sole explanation for the defect: the rescue of the α/β imbalance obtained by the transgenic expression of γ-globin is not sufficient to rescue hemolysis, thus pointing to additional roles for KLF1 (Perkins et al., 2000). In 2015, the first case of severe neonatal anemia with kernicterus due to *KLF1* compound heterozygosis was described in man (Magor et al., 2015), with an erythroid phenotype largely mirroring that observed in mice: hydrops fetalis, hemolytic anemia, jaundice, hepatosplenomegaly, marked erythroblastosis and high levels of HbF. Another report confirms that in humans, although compatible with life, the loss of KLF1 severely impairs erythropoiesis (Lee et al., 2016).

## Quantitative Mutations of KLF1: Haploinsufficiency/Hypomorphic Alleles

*KLF1* is haplosufficient. The loss of one allele is asymptomatic and only genes particularly sensitive to *KLF1* gene dosage are affected. This is observed in the Lutheran In(Lu) Blood group, where either frameshift mutations, introducing premature termination, or amino acids substitutions in the zinc binding domain, lead to reduced or ineffective KLF1 production (Singleton et al., 2008; Helias et al., 2013). Interestingly, the search for possible mutations in an erythroid TF -that turned out to be KLF1- as a cause of the In(Lu) phenotype came from transcriptomic analyses showing that In(Lu) cells express reduced levels of many erythroid-specific genes associated with red cell maturation, including *BCAM* (encoding for the Lu antigen), *ALAS2*, *HBB*, *SLC4A1*, and *CD44* (Singleton et al., 2008). More recently, extended serological and FACS analysis of In(Lu) samples also revealed a reduced expression of *CD35*, *ICAM4*, and *CD147* (Fraser et al., 2018). Interestingly, in one single case the In(Lu) phenotype has been associated with a GATA1 mutation (X414R) (Singleton et al., 2013).

It is now clear that different KLF1 target genes are differentially sensitive not only to KLF1 levels (when one allele carries an inactivating mutation), but also to the type of KLF1 mutation, making it difficult to clearly separate “quantitative” from “qualitative” effects of KLF1 mutations.

Indeed, KLF1 coordinately regulates the expression of a multitude of red cell specific genes including heme biosynthesis genes [*ALAS2*, *HMBS*, *TFR2* (Singleton et al., 2008)], red cell enzymes [such as pyruvate kinase genes -*PKLR* (Viprakasit et al., 2014)], globins (see below) or cell cycle proteins (Hodge et al., 2006; Pilon et al., 2008; Tallack et al., 2009; Gnanapragasam et al., 2016). Thus, depending on the type of mutation, a specific subset of targets can be affected, leading to a broad spectrum of phenotypes (Perkins et al., 2016).

### The Semi-Dominant Phenotype in Nan (Neonatal Anemia) Mouse and in Human CDAIV

This is particularly evident in the case of the neonatal anemia (Nan) semi-dominant (Nan/+) mouse phenotype (Heruth et al., 2010; Siatecka et al., 2010) and in the phenotype observed in human Congenital dyserythropoietic anemia type IV (CDA IV) (Wickramasinghe et al., 1991; Arnaud et al., 2010; Jaffray et al., 2013; Ravindranath et al., 2018). In the Nan mouse model, the E339D substitution in the second ZnF within the Nan allele, alters Nan-KLF1 binding specificity, resulting in an aberrant transcriptome (Gillinder et al., 2017). The homologous E325K heterozygous mutation in CDA IV patients causes the reduced expression of a subset of KLF1 targets (such as *AQP1* and *CD44*), whereas other targets are normally expressed (such as *BCAM*) (Singleton et al., 2011). In analogy with the Nan mouse mutation, it is likely that also in man the E325K mutation could alter the mutant-KLF1 DNA-binding specificity, resulting in detrimental gain of function effects. On the basis of the different charge of the variant residues (Aspartic Acid or Lysine) it is possible to speculate that subsets of targets can be differentially affected by the different mutant proteins, likely explaining the distinct human and mice pathologies (Arnaud et al., 2010; Siatecka et al., 2010). On the other hand, traits common to mouse and human phenotypes could likely result from the reduced (50%) WT-KLF1.

### The Intricate Link Between KLF1, Globin Expression and the Hemoglobin Switching: Direct and Indirect Effects

KLF1 was originally identified by its ability to bind to the β-globin promoter (Miller and Bieker, 1993) and the connection between KLF1 and β-thalassemia is demonstrated by the paradigmatic -87 mutation in the β-globin promoter CACC box (Feng et al., 1994).

Accordingly, the more evident phenotype of *KLF1* knockout mice is a marked β-thalassemia associated with increased *HBG1/HBG2*, suggesting that KLF1 interferes at different levels with globin genes expression. Indeed, the ablation of KLF1 perturbs the 3-dimensional conformation of the β-globin locus (Noordermeer and de Laat, 2008; Schoenfelder et al., 2010). Moreover, mutations creating *de novo* KLF1 motifs can also alter the relative expression within the β-locus: this is the case of the -198 mutation in the γ-promoter that introduces a new KLF1 binding site, generating the British type HFPH (Wienert et al., 2017). Besides these direct effects of loss or gain of KLF1 binding, an intricate network of indirect effects downstream to KLF1 haploinsufficiency/mutations must be considered. Borg et al. (2010) reported a Maltese family with HPFH and mild hypochromatic microcytic RBCs, caused by the KLF1 K288X non-sense mutation, ablating the DNA binding domain. Transcription profiling and functional studies in cells from these subjects revealed low levels of BCL11a, the most important known *HBG1*/*HBG2* repressor, suggesting that failure to properly activate BCL11a is the major cause of the observed HPFH (Borg et al., 2011). This was proven true also in the KLF1-deficient mouse model (Zhou et al., 2010). However, the situation is far more complicated: in another family described shortly thereafter, KLF1 haploinsufficiency did not result in HPFH (Satta et al., 2011). Instead, in this family, HPFH was observed only in compound heterozygotes (non-sense S270X and K332Q missense mutations) together with increased red cell protoporphyrin, a trait observed in the Nan mouse phenotype. Large-scale screening of patients with hemoglobinopathies of different ethnic origin supported the association of *KLF1* mutations with elevated HbF, thus confirming that KLF1 variants are an important source of HbF variation (Gallienne et al., 2012). Finally, more subtle effects of *KLF1* polymorphisms also account for an appreciable proportion of cases with borderline elevated HbA_2_ (Perseu et al., 2011). Thus, again, the pleiotropic effects of KLF1 are the sum of quantitative and qualitative effects, possibly in combination with other genetic modifiers.

## Conclusion and Perspectives

The recent identification of mutations/variants alleles associated with RBC traits involving TFs has greatly increased thanks to new technologies and is expected to further increase in the next few years. This will help not only to explain so far unexplained diseases -and possibly to envisage new therapeutic strategies-, but also to better understand the structure and function of TFs themselves and their involvement in the different gene regulatory networks. This, in turn, will shed light on the contribution of TFs and their target sequences as a source of genetic variability underlying the wide spectrum of the observed erythroid phenotypes.

## Author Contributions

AR conceived and wrote the manuscript. GB, CF, and JS contributed with ideas and discussion. CF created figures.

## Conflict of Interest Statement

The authors declare that the research was conducted in the absence of any commercial or financial relationships that could be construed as a potential conflict of interest.
